# A Smartphone Colorimetric Sensor Based on Pt@Au Nanozyme for Visual and Quantitative Detection of Omethoate

**DOI:** 10.3390/foods11182900

**Published:** 2022-09-18

**Authors:** Biao Zhang, Ruofan Zhou, Huiqi Zhang, Danfeng Cai, Xiaodong Lin, Yihan Lang, Yulou Qiu, Xuping Shentu, Zihong Ye, Xiaoping Yu

**Affiliations:** 1 Zhejiang Provincial Key Laboratory of Biometrology and Inspection & Quarantine, College of Life Sciences, China Jiliang University, Xueyuan Street, Xiasha Higher Education District, Hangzhou 310018, China; 2 College of Food and Bioengineering, Zhengzhou University of Light Industry, Zhengzhou 450001, China

**Keywords:** colorimetric sensor, Pt@Au nanozyme, visualized biosensing, smartphone-based quantitative detection, omethoate

## Abstract

A smartphone colorimetric sensor based on the Pt@Au nanozyme was successfully developed for the visual and quantitative detection of omethoate in fruit and vegetables. The anti-omethoate antibody was conjugated on the surface of the Pt@Au nanozyme as a catalytic functional signal probe, and coating antigen conjugated on the surface of magnetic polystyrene microspheres (MPMs) was used as a separation capture probe. In the sensing system, when the catalytic functional signal probe was combined with a separation capture probe containing no omethoate, the visible blue color appeared with the addition of tetramethylbenzidine (TMB) chromogenic solution, and the maximum B value of the sensing system was obtained via the smartphone. With increasing concentrations of omethoate, the visualization of the sensing system decreased, and the B-value obtained via the smartphone dropped. Under optimal detection conditions, the omethoate could be detected in a linear range of 0.5–50 μg/L (R^2^ = 0.9965), with a detection limit of 0.01 μg/L. The accuracy and reliability of the detection results of this colorimetric sensor were successfully confirmed by enzyme linked immunosorbent assay (ELISA) and gas chromatography. This colorimetric sensor provides a technical reference and potential strategy for the immunoassay of hazard factors in resource-scarce laboratories.

## 1. Introduction

Omethoate, as an organophosphorus pesticide, plays a very important role in improving the yield and quality of edible agricultural products [1]. Omethoate, as a highly efficient and toxic organophosphorus insecticide and acaricide, has mainly been used to effectively control various pests of cotton, wheat, fruit trees, vegetables, sorghum and other crops [2,3,4,5]. However, in order to pursue higher economic benefits, omethoate has been used unreasonably by some producers, and it can be sucked into the plant by the stems and leaves, and transmitted to various parts of the plant, which can easily result in omethoate residues [6,7,8]. Omethoate residue is difficult to degrade via ordinary microorganisms, and after ingestion by consumers, omethoate can inhibit the activity of acetylcholinesterase, affecting the function of human muscles and glands [9]. Meanwhile, omethoate also has serious impacts on food safety, the biological environment, and international trade [10,11,12]. According to GB 2763-2021 Maximum Residue Limits of Pesticides in Food, the allowable daily intake (ADI) of omethoate is 0.0003 mg/kg, the maximum allowable amount of residues in fruits, vegetables, cottonseed and wheat grains is 0.02 mg/kg, and the maximum allowable residue in soybean, sugar beet, sugarcane, tea, dry grain and multigrain cereals is 0.05 mg/kg [13]. Therefore, it is necessary to carry out strict testing, and control the usage and residual amounts of omethoate in accordance with regulations to reduce safety risks.

Many frequently used analytical approaches for omethoate have been reported, such as thin-layer chromatography (TLC) [14], high-performance liquid chromatography (HPLC) [15], gas chromatography (GC) [16], gas chromatography coupled with mass spectrometry (GC/MS) [17], and liquid chromatography coupled with mass spectrometry (LC-MS/MS) [18]. Although the above-mentioned mass spectrometry-related analytical methods are accurate, they have the disadvantages of being time consuming, high inspection and maintenance costs, and requiring professionals for operation [19]. Colorimetric sensors have been reported because of their high accuracy in the visual detection of target analytes without the use of complex equipment [20]. Many colorimetric sensors based on precious nanozymes have been prepared for the visual detection of organophosphorus pesticides, but the human eye is not sensitive to monochromatic or limited color changes, which also limits the application of colorimetric sensors in omethoate detection [21]. Therefore, it is necessary to develop a simple, visual, and quantitative colorimetric sensor based on a portable and inexpensive reading device for the detection of omethoate.

As natural biocatalysts, enzymes have high specificity and efficiency, and play important roles in biological processes such as signal transduction, metabolism, and digestion [22]. However, the production cost of natural enzymes is high, they are easy to denature and inactivate, and the yield is low, making them difficult to apply [23]. Therefore, researchers have gradually developed nanozymes with biomimetic enzyme capabilities and nanoscale dimensions [24]. The developed nanozymes have good stability, low production costs, and simple preparation and purification processes. At present, nearly a thousand nanomaterials with different compositions have been found to have enzyme-like activities. So far, a large number of nanomaterials, such as metal nanoparticles, metal oxide nanoparticles, carbon-based nanomaterials, and metal–organic framework materials, have been shown to have excellent catalytic properties for functioning like natural enzymes [25]. They have been widely used in cancer diagnosis and treatment, food safety detection, environmental monitoring, and other fields [26,27,28]. Therefore, compared with natural enzymes, the colorimetric sensor for omethoate based on the significant advantages of nanozymes are worth exploring.

In this work, the anti-omethoate antibody was conjugated on the surface of the catalytic functional Pt@Au nanoparticle to prepare a signal probe (Pt@Au@anti-omethoate antibody). A colorimetric sensor based on a signal probe and a smartphone was developed for the visual and quantitative detection of omethoate. Meanwhile, the specificity, practicability, and stability of the developed colorimetric sensor were evaluated. The colorimetric sensor is able to achieve the rapid, highly sensitive and low-cost detection of omethoate residue, providing a useful tool for the detection of omethoate residue in edible agricultural products, thus promoting food safety.

## 2. Materials and Methods

### 2.1. Reagents and Instruments

Omethoate, K_2_PtCl_4_, HAuCl_4_, Pluronic F127, EDC, NHS and bovine serum albumin (BSA) were purchased from Sigma-Aldrich (Saint Louis, USA). Dimethoate, dichlorvos, methyl parathion, glyphosate, malathion, profenofos, acetamiprid, trichlorfon were obtained from Aladdin Biochemical Technology Co., Ltd. (Shanghai, China). Anti-omethoate monoclonal antibody was purchased from Kejie Industrial Development Co., Ltd. (Shenzhen, China). The Spotxel®Reader was obtained from the App Store (Sicasys Software GmbH, Germany).

### 2.2. Preparation of Pt@Au Nanozymes, Signal Probe and Capture Probe

Pt@Au nanoparticle nanozymes were synthesized using the liquid phase method with some modifications [29]. Firstly, 9.0 mL of 20 mM K_2_PtCl_4_ solution, 1.0 mL of 20 mM HAuCl_4_ solution, and 104.0 mg of Pluronic F127 were added into a 25 mL round-bottomed flask, and the mixed solution was sonicated to dissolve completely. Then, 1.0 mL of 100 mM ascorbic acid (Vc) was added into the above solution. The mixed solution was sonicated in a water bath at 30 °C for 3 h. The mixed reaction solution was separated by centrifugation at 10,000 rpm/min for 10 min, and the precipitate was washed using ethanol and deionized water. Finally, Pt@Au nanozyme precipitate was reconstituted with 0.9 mL of deionized water and stored at 4 °C until use.

The Pt@Au@Antibody was prepared as follows. Briefly, 50 μg of anti-omethoate antibody was added to 3.0 mL of 15-fold-diluted Pt@Au solution with pH 9.0, and the mixed solution was incubated at 4 °C for 4 h. Then, 30 μL of PBS (0.01 M, pH 7.4, containing 10% BSA) was added to block the excess sites on the surface of Pt@Au. The solution was centrifuged at 10,000 rpm/min for 20 min to remove unconjugated anti-omethoate antibody and BSA. Finally, the Pt@Au@Antibody of the catalytic signal probe was obtained by reconstitution in 1.0 mL of PBS (0.01 M, pH 7.4, 10% BSA) and stored at 4 °C for later use.

The capture probe was prepared as follows. Briefly, 10.0 mg EDC and 5.0 mg NHS were added into 1.0 mL of 5.0 mg/mL magnetic polystyrene microspheres (MPMs) to activate the carboxyl groups on the surface. Then, 50 μg of omethoate coating antigen (OCA) was added into the solution to incubate for 1 h, and 30 μL phosphate buffer saline (PBS) (0.01 M, pH 7.4, containing 10% BSA) was used to block excess MPM epitopes. The supernatant was discarded by external magnetic force, and the conjugation of coating antigen and MPMs (OCA@MPMs) was washed. Finally, the capture probe was prepared by reconstituting the precipitate into 5.0 mL PBS (0.01 M, pH 7.4).

### 2.3. Development of Colorimetric Sensor

The construction process of the colorimetric sensor based on a smartphone was as follows. For the detection system, 50 μL of the capture probe and 50 μL of omethoate standard solution (0, 0.05, 0.1, 0.5, 1.0, 5.0, 10, 50, 100, 200, 500 μg/L) were mixed in a 1.5 mL tube. Then, 50 μL of the catalytic signal probe was added, and the solution was incubated for 20 min at room temperature. The supernatant was discarded by magnetic separation, and the immunoprecipitated complex (Pt@Au@Antibody-OCA@MPMs) was washed with PBS (0.01 M, pH 7.4) in the tube. In addition, 400 μL of TMB chromogenic solution was added into the above tube, and the color reaction time was 5 min at room temperature. The B value of the detection system was captured using Spotxel®Reader on the smartphone, and a linear equation was constructed according to the relationship between the B value and the concentration of the omethoate standard solution. Finally, the specificity and applicability of the colorimetric sensor was evaluated by using structural analogs and similar substances, and actual samples testing, respectively.

### 2.4. Sample Preparation

The samples were pretreated for the developed colorimetric sensor and ELISA according to the national standard GB/T 23200.116-2019 with some modification. Briefly, 20.00 g of Chinese cabbage and small Chinese cabbage samples were put into 150 mL beakers with 40 mL of acetonitrile respectively. The samples were homogenized at high speed for 3 min and the mixture was filtered into a 100 mL centrifuge tube with 6.50 g sodium chloride. The mixed sample solution was vigorously shaken for 2 min and let stand for 30 min. Then, 10 mL of the supernatant was pipetted into a colorimetric tube and dried with nitrogen. One milliliter of pH = 7.0 PBS buffer solution containing 5% methanol was used to dissolve the residue, and the solution was filtered with a microporous membrane (0.22 × 25 mm) for later analysis. For GC analysis, the other steps are basically the same as the above, except that 5.0 mL of acetone was used to reconstitute the sample residue after nitrogen blowing, and then the 25-fold diluted sample solution was measured.

### 2.5. ELISA and GC Analysis

The detailed procedure of ELISA for omethoate was listed in Supplementary Material. The omethoate concentration in samples was measured by GC 2010 Plus equipped with flame photometric detector. Then, 50% of polyphenylmethylsiloxane quartz capillary column (30 m × 0.53 mm (inner diameter) × 1.0 μm) was employed for the detection of omethoate. The applied parameters for the detection conditions were: column temperature, 150 °C for 2 min, 8 °C/min programmed temperature increase to 210 °C, 5 °C/min programmed temperature increase to 250 °C, maintained for 15 min, with nitrogen as the carrier gas at a flow rate of 8.4 mL/min. The temperatures of the injection port and the detector were 250 °C and 300 °C, respectively. The injection volume was 1.0 μL.

## 3. Results and Discussion

### 3.1. Principle of the Colorimetric Sensor

**Scheme 1:**
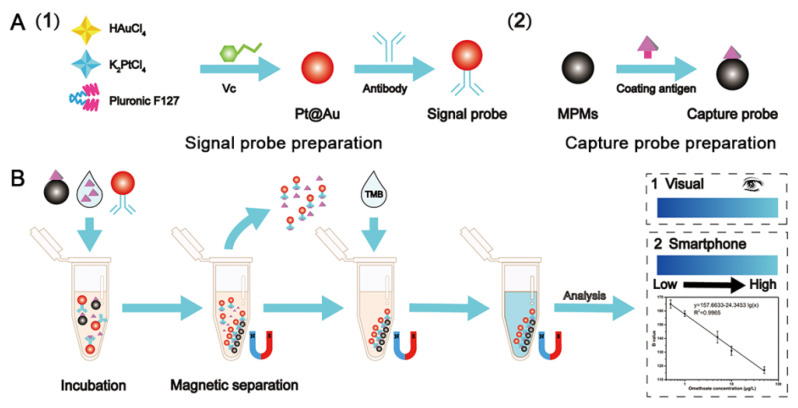
Schematic illustration of preparation of signal probe and capture probe (**A**), and the detection process of proposed colorimetric sensor (**B**).

The preparation of the signal probe, capture probe and detection process is shown in Scheme 1. K_2_PtCl_4_, HAuCl_4_, Pluronic F127 and Vc were employed to prepare the Pt@Au nanozymes, and MPMs and coating antigen of omethoate were used to prepare the capture probe (Scheme 1A). The capture probe, the omethoate standard (or sample) solution, and the signal probe are dropped into a 1.5 mL centrifuge tube and incubated for 20 min. As shown in Scheme 1B, the supernatant containing the Pt@Au@antibody-omethoate (or Pt@Au@antibody) was discarded under external magnetic force. Then, TMB solution was added into the tube containing the immunoprecipitated complex (Pt@Au@Antibody-OCA@MPMs). In the absence of omethoate molecules, the color visible to the naked eye was the bluest, and the B value based on the smartphone reading was the highest. With the increase of omethoate concentration in the detection system, the less signal probes captured by the capture probe, the visible color by the naked eye was lighter, and the B value obtained by the smartphone was relatively lower. According to the obtained B value and the concentrations of omethoate, the quantitative detection standard curve for omethoate was constructed.

### 3.2. Characterization of Pt@Au Nanozyme

**Figure 1:**
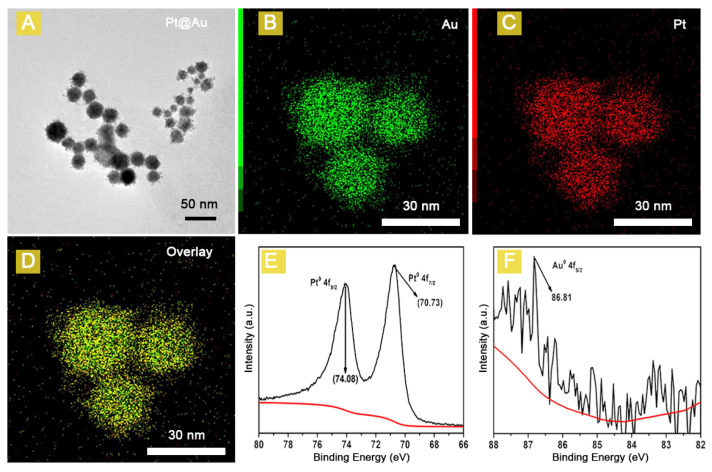
TEM images of Pt@Au (**A**); elemental mapping images of Au (**B**), Pt (**C**), and Au and Pt (**D**) in Pt@Au nanoparticles; and high-resolution XPS spectra of Pt4f (**E**) and Au4f (**F**).

In this work, Pt@Au nanoparticles with catalytic function were prepared using the liquid phase method. As shown in Figure 1A, the Pt@Au nanoparticles are spherical in Pt@Au TEM image, and the average particle size of Pt@Au Pt@Au nanoparticles is 19.0 ± 5.6 nm, on the basis of statistical analysis. The distribution of Au and Pt elements was analyzed, and elemental mapping images (Figure 1B–D) show the distribution of Au and Pt elements in Pt@Au nanoparticles. The uniform distribution of Pt and Au elements shows that Pt@Au nanoparticles have both the catalytic function of Pt and the ability of Au coupled to antibodies. The XPS spectra of Pt 4f and Au 4f are shown in Figure 1E,F, the binding energy peaks at 70.73 eV and 74.08 eV correspond to Pt0 4f_7/2_ and Pt0 4f _5/2_, and the peak at 86.81 eV reflects Au0 4f_5/2_. The results are basically consistent with those reported in the literature [30], which indicates that the Pt@Au nanoparticles had been successfully prepared. Therefore, Pt@Au nanoparticles can be applied to prepare the catalytic signal probe, based on which the colorimetric sensor for omethoate was constructed.

### 3.3. Development of Colorimetric Sensor

**Figure 2:**
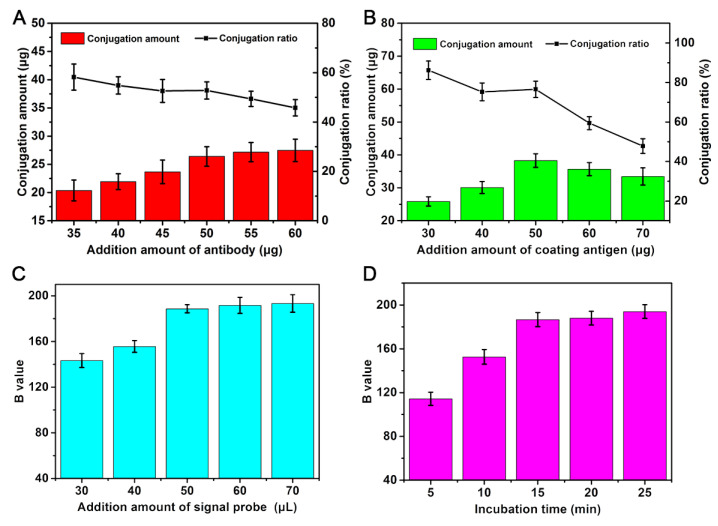
The optimization of the working parameters of the colorimetric sensor. (**A**) Added amount of anti-omethoate antibody, (**B**) added amount of coating antigen, (**C**) added amount of signal probe, and (**D**) incubation time.

To obtain better detection performance with the colorimetric sensor based on a smartphone, the amount of anti-omethoate antibody added in the preparation of signal probes, the amount of coating antigen added in the preparation of the capture probes, the amount of signal probe added, and the incubation time were optimized. The amounts of added anti-omethoate antibody and coating antigen were optimized by the conjugation amount using the BCA protein quantification kit. The signal probe and incubation time were optimized based on the B value. The results are shown in Figure 2, and the optimal dosages of anti-omethoate antibody and coated antigen were 50 μg and 50 μg, respectively, and the corresponding conjugation ratios were 52.80% and 76.60%. Under the above conditions, two probes were prepared for the colorimetric sensor. According to the B value of the detection system, the optimal added amount of Pt@Au@Antibody, and the incubation time for the development of the colorimetric sensor are 50 μL and 15 min, respectively.

**Figure 3:**
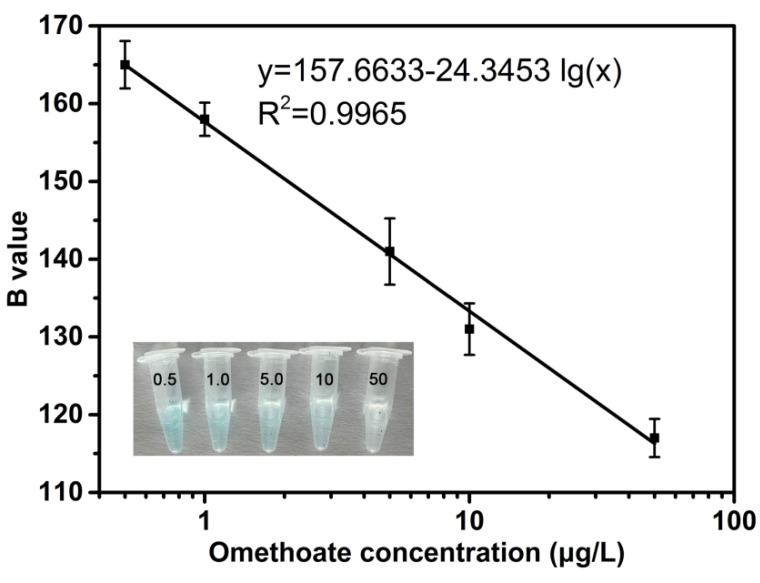
The standard curve of the developed colorimetric sensor for omethoate; and inset: color development images at omethoate concentrations of 0.5~50 μg/L.

Under the above experimental conditions, the omethoate standard solution with different concentrations (0, 0.05, 0.1, 0.5, 1.0, 5.0, 10, 50, 100, 200, 500 μg/L) was detected by the developed colorimetric sensor. The immunoprecipitated complex (Pt@Au@Antibody-OCA@MPMs) was separated by external magnetic force and 400 μL of TMB mixture solution was added to the above complex system. After color reaction for 5 min, the B value of the detection system was obtained in a dark box using the SpotxelRReader software. The dark box device was prepared by transforming an LED photo studio (Figure S1). There is a circle of lights on the top of the dark box device, and the lights can be lit by a mobile power supply or a socket power supply. As shown in Figure 3, the B value shows a decreasing trend with increasing omethoate concentration (0.5, 1.0, 5.0, 10, 50 μg/L), which is due to the competition between omethoate molecules and the capture probe with respect to binding to the catalytic signal probe. The visual detection results regarding the presence of different omethoate concentrations (0, 0.05, 0.1, 0.5, 1.0, 5.0, 10, 50, 100, 200, 500 μg/L) are shown in Figure S2 in the Supplementary Materials. The linear detection equation for omethoate is y = 157.6633-24.3453 lg(x) (R^2^ = 0.9965), and the linear detection range is 0.5 to 50 μg/mL, with a detection limit of 0.01 μg/mL. To evaluate the sensitivity of the developed colorimetric sensor under the working conditions (Table S1), the same anti-omethoate antibody and coating antigen were employed to establish traditional indirect competitive ELISA for the detection of omethoate. The standard inhibition curve of the ELISA for omethoate is shown in Figure S3. The limits of detection (IC_15_) and sensitivity (IC_50_) of ELISA for omethoate are 5.54 μg/L and 131.17 μg/L, respectively. Compared with the ELISA for omethoate, the sensitivity of the developed colorimetric sensor is improved by two orders of magnitude. Compared with the other methods for detecting omethoate presented in the literature [31,32,33,34], this proposed colorimetric sensor improves the specificity and detection speed for omethoate (Table S2).

### 3.4. Evaluation of Specificity 

**Figure 4:**
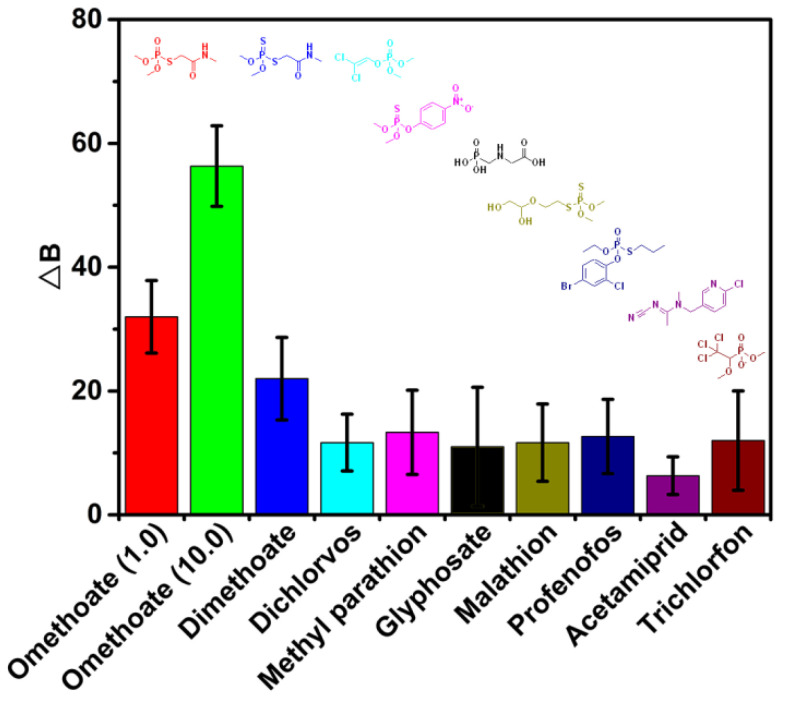
Specificity analysis of the developed colorimetric sensor.

The evaluation of specificity is an important indicator for an immunoassay. In this work, dimethoate, dichlorvos, methyl parathion, glyphosate, malathion, profenofos, acetamiprid, and trichlorfon, as functional and structural analogs of omethoate, were employed to evaluate the specificity of the colorimetric sensor. As shown in Figure 4, the ΔB was 15 and 75 when the concentrations of omethoate were 0.1 μg/L and 10 μg/L, and the difference in the B value (ΔB) between the sample without omethoate and 1000 μg/L of different analogs of omethoate, respectively. The inset is the structure diagram of the corresponding structural analog of omethoate. Meanwhile, the ΔB values for dimethoate, dichlorvos, methyl parathion, glyphosate, malathion, profenofos, acetamiprid, and trichlorfon were less than 10. These results indicate that the anti-omethoate antibody specifically recognized omethoate, and that the constructed colorimetric sensor, with a good specific recognition for omethoate, would not be affected by the functional and structural analogs of omethoate. The constructed colorimetric sensor is able to analyze omethoate with high specificity in real samples.

### 3.5. Analytical Application in Food Samples

To evaluate the practicability and stability of the developed colorimetric sensor, we selected Chinese cabbage and tomato to measure the background concentration and the different concentration levels of the added omethoate in actual samples. The samples were spiked with three concentration levels of omethoate: 20 μg/kg, 200 μg/kg, and 1000 μg/kg. The results obtained when detecting omethoate in Chinese cabbage and tomato using the developed colorimetric sensor were confirmed by GC and ELISA based on the same antibody and coating antigen of omethoate. As shown in Table 1, the concentrations of omethoate in the non-spiked Chinese cabbage and tomato were not detected using the developed method, ELISA, or GC. With the addition of omethoate into Chinese cabbage at concentrations of 20 μg/kg, 200 μg/kg, and 1000 μg/kg, the results detected by the developed assay were 17.67 μg/kg, 178.83 μg/kg and 902.00 μg/kg, respectively, and the results detected by ELISA (or GC) were 18.40 μg/kg, 177.97 μg/kg, and 895.60 μg/kg (or 19.03 μg/kg, 189.50 μg/kg, and 905.20 μg/kg). The same level gradient concentrations of omethoate were added into the tomato, and the detection results using the developed assay were 17.70 μg/kg, 172.30 μg/kg and 803.47 μg/kg, and the detection results using ELISA (or GC) were 17.53 μg/kg, 168.83 μg/kg, and 854.80 μg/kg (or 17.51 μg/kg, 177.83 μg/kg, and 897.79 μg/kg), respectively. The above results show that the results detected by the developed colorimetric sensor are in good agreement with those detected by GC and ELISA (R > 0.99). The stability of the results detected using the developed colorimetric sensor was evaluated using the coefficient of variation (CV). The CV value of the colorimetric sensor (5.16–11.47%) is slightly larger than that of ELISA (4.10–6.19%) and GC (2.12–4.33%), indicating that the accepted stability of the colorimetric sensor is not as good as that of ELISA and GC, and subsequent work can focus on improving the stability of the colorimetric sensor. Meanwhile, the colorimetric sensor could also be a selective tool for the detection of omethoate in fruits and vegetables.

## 4. Discussion

In this work, the anti-omethoate antibody conjugated to the surface of the prepared Pt@Au nanozyme as a catalytic signal probe was used to construct a colorimetric sensor. The developed colorimetric sensor based on the smartphone was able to accurately and rapidly detect omethoate in Chinese cabbage and tomato. Meanwhile, the constructed colorimetric sensor could be used to perform semi-quantitative visual detection of omethoate by means of the naked eye, without sophisticated detection instruments. The constructed colorimetric sensor has a linear detection of omethoate in the range of 0.5–50 μg/L, with a detection limit of 0.01 μg/L. The accuracy and reliability of this colorimetric sensor was successfully confirmed by ELISA and GC, and the detection results obtained by this colorimetric sensor are in good agreement with the results detected using ELISA and GC. In conclusion, a convenient, intelligent, and highly sensitive detection method for omethoate is presented in this work, demonstrating that a strategy based on smartphone and Pt@Au nanozyme could be successfully applied to the detection and monitoring of pesticide residues in other fruits and vegetables.

## Figures and Tables

**Table 1:** Detection results of omethoate in Chinese cabbage and tomato (*n* = 10).

Samples	Added (μg/kg)	This Colorimetric Sensor			ELISA			GC		
		Detected (μg/kg)	Recovery (%)	CV (%)	Detected (μg/kg)	Recovery (%)	CV (%)	Detected (μg/kg)	Recovery (%)	CV (%)
Chinese cabbage	0	ND	-	-	ND	-	-	ND	-	-
	20	17.67	88.33	10.92	18.40	92.00	4.10	19.03	95.17	2.12
	200	178.83	89.42	9.61	177.97	88.98	6.02	189.50	94.75	3.71
	1000	902.00	90.20	5.16	895.60	89.56	6.19	905.20	90.52	4.31
Tomato	0	ND	-	-	ND	-	-	ND	-	-
	20	17.70	88.50	11.47	17.53	87.67	5.71	17.51	87.57	4.33
	200	172.30	86.15	8.13	168.83	84.42	5.78	177.83	88.92	3.37
	1000	803.47	80.35	6.02	854.80	85.48	6.15	897.79	89.78	3.23

CV, coefficient of variation; ND, not detected; -, not be calculated.

## Data Availability

The datasets generated for this study are available on request from the corresponding author.

## Supplementary Materials

The following supporting information can be downloaded at: https://www.mdpi.com/article/10.3390/foods11182900/s1, Figure S1: the dark box device diagrams in different locations; Figure S2: visual results for the different concentrations of omethoate; Figure S3: standard inhibition curve of the ELISA for omethoate in PBS; Table S1: optimal working conditions of ELISA; Table S2: Comparison of sensors for omethoate detection.
