# Supplementary material for: A Smartphone Colorimetric Sensor Based on Pt@Au Nanozyme for Visual and Quantitative Detection of Omethoate
Source: Foods. 2022 Sep 18;11(18):2900. doi: 10.3390/foods11182900 (PMC9498264; doi:10.3390/foods11182900)
Supplement: Supplementary file 1 [file foods-11-02900-s001.zip › foods-1906119-supplementary.pdf]

A Smartphone Colorimetric Sensor Based on Pt@Au Nanozyme for Visual and Quantitative Detection of Omethoate

Biao Zhang [1,*], Ruofan Zhou [1], Huiqi Zhang [1], Danfeng Cai [1], Xiaodong Lin [2], Yihan Lang [1], Yulou Qiu [1], Xuping Shentu [1], Zihong Ye [1] and Xiaoping Yu [1]

[1] Zhejiang Provincial Key Laboratory of Biometrology and Inspection & Quarantine, College of Life Sciences, China Jiliang University, Xueyuan Street, Xiasha Higher Education District, Hangzhou 310018, China
[2] College of Food and Bioengineering, Zhengzhou University of Light Industry, Zhengzhou 450001, China
* Correspondence: zb@cjlu.edu.cn; Tel.: +86-136-2219-9129

Contents

Supplementary experimental method

Table S1 Table S2

Figure S1 Figure S2 Figure S3

Development of Indirect Competitive ELISA

The coating antigen solution (100 μL/well) was added into a 96-well plate and incubated at 4 °C overnight. After washing three times with PBST (10 mmol/L PBS with 0.05% Tween-20), the microwells were blocked by addition of the blocking solution (200 μL/well) at 37 °C for 1 h. After the microwells were washed, omethoate standard or sample solution (50 μL/well) and antibody in PBS (50 μL/well) were added into each well, and then the microwell plate was incubated at 37 °C for 1 h. After washing, the HRP-labeled secondary antibody solution (100 μL/well) was added into each well and then the microwell plate was incubated at 37 °C for 30 min. After washing again, TMB substrate solution (100 μL/well) was added into micro-wells and developed for 15 min at 37 °C. The reaction of each well was stopped by addition of 50 μL H_2SO_4 (1.25 mol/L). Finally, the absorbance was measured in a plate reader with the test wavelength at 450 nm.

Table S1 Optimal working conditions of ELISA

factor	omethoate
amount of coating antigen	0.10 μg per well
dilution factor of antibody	1:16000
blocking solution	0.5% skim milk powder
dilution factor of HRP-labeled secondary antibody	1:10000

Table S2 Comparison of sensors for omethoate detection

Methods	Linear Range	Detection Limit	Selectivity	Time	Reference
Colorimetric	1.3-100 μmol/L	1.3μmol/L	No	32 min	[1]
Colorimetric	0.1-10 μg/mL	0.1 μg/L	No	12 min	[2]
Visual screening card	0.1-10 μg/mL	0.1 μg/L	No	25 min	[3]
SERS	24-500 μmol/L	24 μmol/L	Reacted to 4 OPs	21 min	[4]
Colorimetric	0.5-50 μg/L	0.01 μg/L	Only for omethoate	20 min	This work

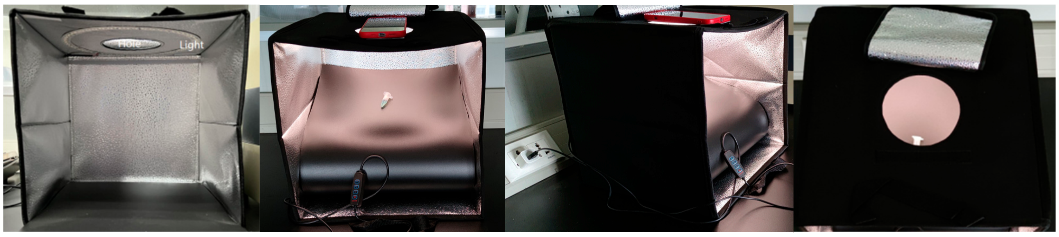

Figure S1 The dark box device diagrams in different locations

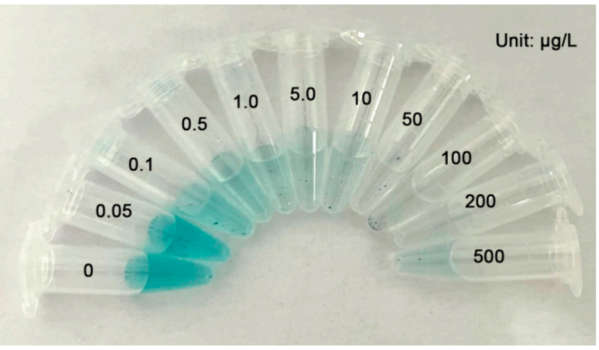

Figure S2 Visual results for the different concentrations of omethoate.

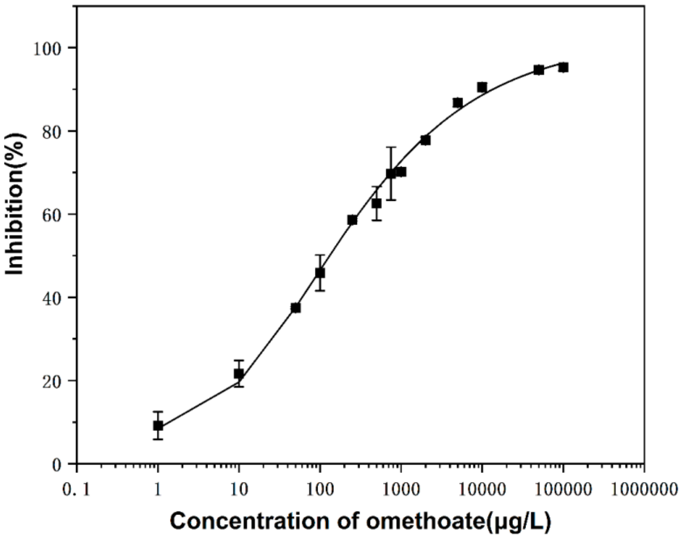

Figure S3 Standard inhibition curve of the ELISA for omethoate in PBS.

References

1. Oujji NB, Bakas I, Istamboulie G, Ait-Ichou I, Ait-Addi E, Rouillon R, Noguer T. Food Control, 2014, 46: 75-80.
2. Han Z, Chi CS, Bai, B, Liu G, Rao QX, Peng SJ, Liu H, Zhao ZH, Zhang DB, Wu AB. Anal Chim Acta, 2012, 720: 126-133.
3. Guo XS, Zhang XY, Cai Q, Shen T, Zhu SM. Food Control, 2013, 30:15-23.
4. Pang S, Labuza TP, He L. Analyst, 2014, 139: 1895-1901.
